# The role of the sound of objects in object identification: evidence from picture naming

**DOI:** 10.3389/fpsyg.2014.01139

**Published:** 2014-10-08

**Authors:** Claudio Mulatti, Barbara Treccani, Remo Job

**Affiliations:** ^1^Dipartimento di Psicologia dello Sviluppo e della Socializzazione, Università degli Studi di PadovaPadova, Italy; ^2^Dipartimento di Storia, Scienze dell'Uomo e della Formazione, Università degli Studi di SassariSassari, Italy; ^3^Dipartimento di Psicologia e Scienze Cognitive, Università degli Studi di TrentoRovereto, Italy

**Keywords:** picture naming, object recognition, grounded cognition, embodied cognition, language, semantics, object processing, sound representation

## Abstract

In the present work we were concerned with the role of sound representations in object recognition. In order to address this issue we made use of a picture naming task in which target pictures might be accompanied by a white-noise burst. White-noise was thought to interfere with the representation of the sound possibly associated with the depicted object. We reasoned that if such a representation is critical for the recognition of objects strongly associated with certain sounds, white-noise interference should affect the naming of pictures representing objects with typical sounds leaving the naming of object without typical sounds unaffected. The results were congruent with the predictions and consistent with a view of the semantic representations of objects as collection of related representations, modal in nature, and mandatorily accessed.

## Introduction

This study deals with the role of sounds in object recognition in humans. Indeed, some objects are easily associated with a sound, i.e., some objects possess either a *typical sound* or category of sounds. This is the case, for example, of objects such as “bell” or “motorbike.” Other objects do not possess typical sounds or can be associated with particular sounds only with difficulty. This is the case, for example, of objects such as “table” or “pillow.”

Given that objects can be classified as a function of whether they possess or not a typical sound, a legitimate question is whether the typical sounds play any role in the visual recognition of the related objects. There are at least two opposed scenarios to frame this question.

In the first scenario, upon the presentation of a visual object the system first accesses an abstract representation of that object and then—depending on the task at hand—accesses the representations of information related to that object: among these representations is the representation of the typical sound. Thus, in this scenario, the access to the typical sound is post-categorical, in the sense that the object is first recognized as an instance of a particular kind (e.g., a “dog”) and then the related information is retrieved (cf. Allport, 1977; Mulatti et al., 2014). Here, the typical sound may be activated but, since its retrieval follows the identification of the object, it does not play any role in the recognition of the object.

In the second scenario, all stored representations associated to a given object are immediately and mandatorily activated upon the visual presentation of an instance of that kind of object. Here, the identification of the object does not consist in the activation of an abstract semantic representation of this object but instead corresponds to the activation of all stored representations. In other words, object identification *is* the activation of object knowledge. For objects with a typical sound, the typical sound is part of the knowledge of that object and, therefore, the activation of the typical sound is part of the process of object identification: an object cannot be identified without its typical sound being activated. Thus, in this second scenario the access to the typical sound is pre-categorical and has a functional significance in the identification process: typical-sound activation does not only occur when it is requested by the task and it is not simply a concomitant, epiphenomenal, effect of the identification (cf. Kiefer and Barsalou, 2013).

These two scenarios can be seen as the two extreme positions of a continuum of scenarios going from post- to pre-categorical, and therefore intermediate positions are possible (Pezzulo, 2011). In this study we attempt to provide evidence in favor of one of these two extremes.

Previous studies investigating cross-modal effects in object recognition have shown that when both visual and auditory information (e.g., the picture of an object and the typical sound of an object) are presented in object recognition tasks, both types of information affect the time need to emit a response: responses are usually faster when participants are presented with cross-modal congruent stimuli (i.e., the sound refers to the object depicted in the picture) than when they are presented with incongruent stimuli (i.e., the sound is typical of another object; e.g., Laurienti et al., 2003). Based on psychophysiological and neuroimaging findings, visual and auditory inputs are thought to interact quite early (i.e., at sensory processing stages; e.g., Giard and Peronnet, 1999). Yet, according to the most accepted view, they would be integrated afterwards (e.g., Hocking and Price, 2008), at higher cognitive processing stages. Sensory information from unimodal processing channels would converge onto a modality–independent semantic system (Coltheart, 1987). Cross-modal semantic congruency effects would arise at this processing level and, consistent with this view, they are typically interpreted within a post-categorical framework (cf., Schneider et al., 2008). Congruent visual and auditory inputs are seen as independent perceptual cues activating the same (amodal) semantic knowledge. The addition of a redundant congruent perceptual cue (e.g., the typical sound of an object when participants has to recognize a picture) can facilitate the recognition of the object by enhancing its activation level (then reducing competition) and is particularly useful when the object has many structurally and semantically similar neighbors that compete for selection (Humphreys et al., 1995). In this respect, a congruent sound does not have any facilitatory role in the recognition of an object when recognition can proceed on the basis of visual stimuli alone (e.g., Hocking and Price, 2008).

However, results of cross-modal integration studies might be equally easily interpreted by a pre-categorical account assigning to sounds a functional role in visual object recognition. Indeed, results obtained in tasks providing for the presentation of both visual and auditory stimuli related to a given object cannot help to discriminate between the two accounts: results of these studies tell us nothing about whether the typical sound of an object is activated even when only the visual form of this object is presented, nor whether the sound activation, possibly triggered by the mere presentation of visual stimuli (e.g., Nyberg et al., 2000), is simply a byproduct of object recognition processes or is critical for, and inextricable from, such processes.

The cross-modal semantic congruency paradigm does not then seem a suitable tool for the investigation of the possible functional role of typical sounds in visual object recognition. In the experiment presented below, participants are administered a visual object recognition task in which the activation of the object typical sound is neither required nor triggered by redundant auditory stimuli: we do not present the typical sound of an object or cues that can somehow evoke such a sound, but rather present stimuli that should interfere with the possible (unrequested) activation of the typical sound induced by the recognition process itself.

In this experiment, participants perform a picture naming task. Our choice of the task fell on picture naming because of two aspects that characterize it. First, picture naming requires access to the semantic system (e.g., Potter and Faulconer, 1975; Mulatti et al., 2010). Second, picture naming does not stress the processing of any particular aspect of the meaning in order to be performed, that is it does not require the retrieval of any particular feature of the meaning (Dell'Acqua et al., 2010; Mulatti and Coltheart, 2012): in the present context this means that the naming of a picture of an object possessing a typical sound does not mandatorily require the activation of sound-related representations. So, if an effect due to the typical sound were found in picture naming, we could reasonably conclude that the representation of the typical sound is mandatorily activated in object recognition because of the architecture of the semantic system and not because of the requirements of the task.

In the study, participants name pictures depicting two kinds of objects, objects possessing typical sounds and objects not possessing typical sounds. Here, possessing or not a typical sound is an operational construct that should not be interpreted literally. An object possesses a typical sound if a sound can be easily associated to that object. An object does not possess a typical sound if no sound can be easily associated to that object.

Each picture is presented twice to each participant, once in each of two conditions. In one condition, the picture is presented along (SOA = 0) with a brief (400 ms) white-noise sound. In the other condition, the picture is presented in isolation, i.e., not accompanied by any sound. White noise should interfere with the retrieval of typical sounds. This is supported by the results of previous studies suggesting the existence of a close link between auditory perception and auditory imagery and memory (e.g., the neural structures active in auditory perception are also active in auditory imagery; see Hubbard, 2010, for a review) and showing that auditory distraction may selectively impair recall of auditory information (e.g., Vredeveldt et al., 2011).

This manipulation then allows us to investigate the possible involvement of typical sound activation in the recognition of the objects depicted in the pictures. If the access to the typical sound is post-categorical, then the concurrent presentation of white noise should not affect the naming of objects with a typical sound more than the naming of objects without a typical sound—and both should not differ from naming the same objects when presented in isolation, i.e., without white noise. This is because picture naming rests on the identification of the object stimulus, and, according to the post-categorical view, the identification of a visual object stimulus precedes—and is independent from—the activation of the representation of the typical sound. So, even if the presence of white-noise affects representation of the sound typically associated with the presented object, this would not affect object naming, regardless of whether the object possesses a typical sound or not.

Instead, if the access to the typical sound is pre-categorical, then the presence of white-noise should interfere more with the naming of objects possessing a typical sounds compared to objects not possessing typical sounds—with respect to the control condition. In the pre-categorical scenario, the activation of the typical sound representation is part of the process of object identification, for those objects that possess a typical sound. Therefore, if the presence of white-noise interferes with the activation of the representation of the typical sound, it also interferes with the identification of the object. Given that object naming rests on object identification, the presence of white-noise should interfere with object naming, but only in the case that the to-be-named object possesses a typical sound.

## Experiment

### Methods

#### Participants

Thirty-two students of the Università degli Studi di Padova voluntarily participated in the experiment. They were all native Italian speakers with normal or corrected-to-normal vision, and none reported auditory impairments. Oral consent was obtained from each participant before the beginning of the experiment as required by the regulation of the ethical committee of the Università degli Studi di Padova regarding behavioral studies involving adult human participants.

#### Design

A 2 Type Of Object (possessing vs. not-possessing typical sound) × 2 Presentation Condition (picture accompanied with white noise vs. alone) within-subject design was used.

#### Material

128 line-drawing (black on white background) pictures of objects (half possessing a typical sound and half not possessing a typical sound) were selected as stimuli. They were taken from the databases of Bates et al. (2003), and of Dell'Acqua et al. (2000). Fourteen participants (not involved in the main study) evaluated how easily each object evocates a typical sound by means of a 7 points Likert-like scale (1 = difficult). In average, objects that were classified as possessing a typical sound received a score of 6.4 (range 5.3–7; *SD* = 0.5) whereas objects that were classified as not possessing a typical sound received a score of 1.7 (range 1–2.6; *SD* = 0.5). Stimuli in the two categories were balanced in terms of frequency of occurrence, name agreement, length, and phonological neighborhood size (*t*s < |1|). The names of the stimuli are reported in the Appendix in Supplementary Material.

A digital hissing sound (44.1 kHz, −6 dBFS) of 400 ms of duration was construed and used as the white-noise stimulus.

#### Apparatus and procedure

The experiment took part in a dim-lit sound attenuated room equipped with a PC to which a 17 in. CRT monitor, a voice key, and a pair of speakers were connected. The experiment was controlled by a software developed in E-Prime 2.0. Participants were tested individually and instructed to name the picture as quickly and accurately as possible. Each trial started with the presentation of a fixation point (+) for 500 ms. At its off-set a picture was presented. Reaction times were time-locked to the onset of the picture. Pictures were presented in a single block and, as a function of the experimental condition, they presented either in isolation or accompanied (SOA = 0) by the white-noise sound which was delivered by the speakers. The order of presentation of the stimuli for each participants was random. Apparatus and naming errors were scored manually by the experimenter. Before the picture naming experiment, participants were familiarized with the pictures and their names. The experimental session was preceded by a 20-trials practice session.

### Results

#### Reaction times (RTs)

Apparatus failures (2.2%) and naming errors (2.8%) were removed prior to RTs analyses. Correct RTs were submitted to the Van Selst and Jolicoeur (1994) recursive outlier trimming procedure, which excluded an additional 2.4% of the data. Mean naming latencies according to conditions are reported in Table 1. In the by-subjects ANOVA (*F*1), both Type Of Object (possessing vs. not-possessing typical sound) and Presentation Condition (picture accompanied with white noise vs. alone) were treated as within-subjects factors. In the by-items ANOVA (*F*2), Type Of Object was treated as a between-items factor whereas Presentation Condition was treated as a within-items factor. The analyses showed a significant main effect of Type of Object in the by-subjects analysis, *F*1_(1, 31)_ = 6.8, MSE = 3640, *p* < 0.05, but not in the by-item analysis, *F*2_(1, 126)_ = 1.4, MSE = 34061, *p* = 0.24, a significant main effect of Presentation Condition, *F*1_(1, 31)_ = 4.4, MSE = 2342, *p* < 0.05, *F*2_(1, 126)_ = 7.5, MSE = 3567, *p* < 0.01, and, crucially, a significant interaction, *F*1_(1, 31)_ = 4.9, MSE = 2854, *p* < 0.05, *F*2_(1, 126)_ = 8.6, MSE = 3567, *p* < 0.005. Planned comparisons revealed that RTs were significantly slower when objects possessing typical sounds were presented with white-noise with respect to when presented alone, *t*-participants_(31)_ = 3.1, *p* < 0.005, *t*-items_(63)_ = 3.8, *p* < 0.001. In contrast, RTs for the objects not possessing typical sounds were unaffected by the presence of white-noise, both *t*s < |1|.

**Table 1:** **Mean reaction times (*RT*s) and percentage of errors (*E*%) according to conditions**.

**Typical sound**	**White noise**				**Differences (*RT*)**
	**Without**		**With**		
	***RT***	***E*%**	***RT***	***E*%**	
With	910	2.9	951	2.3	−41
Without	903	3.1	902	2.9	1

#### Errors

Mean error percentages are reported in Table 1. No effects were significant in the analyses of errors, *F*s < 1.

## Discussion

The present study aimed at assessing the role of sound representation in object recognition. In order to address this issue we have exploited a picture naming task in which target pictures might be accompanied by a white-noise burst. White noise was thought to interfere with the representation of the sound possibly associated with the depicted object. We reasoned that if such a representation is critical for the recognition of objects strongly associated with certain sounds, white-noise interference should affect the naming of pictures representing these objects.

The results are clear cut, as a white-noise burst presented with a to-be-named picture does interfere with picture naming but only if the picture depicts an object possessing a typical sound. There are two aspects of this finding that are worth discussing.

First, in a standard picture naming task participants are only required to name the stimulus they are presented with as quickly as possible, they are *not* required to retrieve particular aspects of the meaning of the stimulus, as its typical color, smell or sound. Thus, the finding that the presentation of white noise interferes with picture naming when the stimulus depicts an object possessing a typical sound suggests that the activation of the auditory representations associated to that object is mandatory upon stimulus presentation.

Second, the fact that the naming of objects possessing a typical sound is interfered with by the concurrent presentation of a white-noise sound-stimulus suggests that the representations of sounds are activated *while* the object is being identified, that is that object-related sound are activated before complete identification of the object had occurred. In other words, this finding is congruent with a pre-categorical view—and therefore incongruent with a post-categorical view—of the access to object-related sound representations, thus suggesting that object-related sound representations participate in object identification.

Once established that the pre-categorical scenario is more congruent with the above finding than a post-categorical scenario, a question naturally arises: why does white-noise interfere? That is, what is the mechanism that causes this interference? One possibility is to assume that auditory representations are *modal*, in the sense that acquired auditory knowledge is stored (at least partially) in the same systems that subserve auditory processing (Kiefer et al., 2008; Vermeulen et al., 2008). Thus, upon the presentation of a visual object possessing a typical sound, the corresponding modal auditory representation—residing in the auditory processing system—is activated. If the system storing auditory knowledge is also the system subserving auditory processing, then the presentation of an auditory stimulus—e.g., white-noise—will interfere with the possible concurrent activation of auditory representations—e.g., the typical sound of the object (see Connell and Lynott, 2012, for a discussion), which is what we observed.

A similar explanation has been proposed by Matheson et al. (2014) to account for the interference effects they found in a task requiring the execution of irrelevant movements while participants named picture of either animals or inanimate objects. Matheson et al. observed that the naming of manipulable artifacts was affected by concurrent motor activity, whereas no effects of motor activity were found when participants named non-manipulable animals. The authors concluded that the same neural sensorimotor networks are involved in encoding and retrieving object knowledge (cf. Barsalou, 1999, 2008) and the concurrent irrelevant motor activities interfered with the activation of motor programs that were necessary to retrieve object knowledge.

In conclusion, our finding supports a pre-categorical view of the semantic of objects and is consistent with a concept of concepts as collections of mandatorily accessed, related representations (Redmann et al., 2014) which are modal in nature.

### Conflict of interest statement

The authors declare that the research was conducted in the absence of any commercial or financial relationships that could be construed as a potential conflict of interest.

## References

[B1] Allport D. A. (1977). On knowing the meaning of words we are unable to report: the effects of visual masking, in Attention and Performance VI, ed Dornic S. (New York, NY: Academic Press), 505–534

[B2] Barsalou L. W. (1999). Perceptual symbol system. Behav. Brain Sci. 22, 577–660 11301525 10.1017/s0140525x99002149

[B3] Barsalou L. W. (2008). Grounded cognition. Annu. Rev. Psychol. 59, 617–645 10.1146/annurev.psych.59.103006.093639 17705682

[B4] Bates E. D'Amico S. Jacobsen T. Szekely A. Andonova E. Devescovi A. (2003). Timed picture naming in seven languages. Psychon. Bull. Rev. 10, 344–380 10.3758/BF03196494 12921412 PMC3392189

[B5] Coltheart M. (1987). Functional architecture of the language-processing system, in The Cognitive Neuropsychology of Language. eds Coltheart M. Sartori G. Job R. (London: Lawrence Erlbaum Associates), 1–26

[B6] Connell L. Lynott D. (2012). When does perception facilitate or interfere with conceptual processing? The effect of attentional modulation. Front. Psychol. 3:474 10.3389/fpsyg.2012.00474 23130012 PMC3487424

[B7] Dell'Acqua R. Sessa P. Peressotti F. Mulatti C. Navarrete E. Grainger J. (2010). ERP evidence for ultra-fast semantic processing in the picture-word interference paradigm. Front. Psychol. 1:177 10.3389/fpsyg.2010.00177 21833238 PMC3153787

[B8] Dell'Acqua R. Lotto L. Job R. (2000). Naming times and standardized norms for the Italian PD/DPSS set of 266 pictures: direct comparisons with American, English, French, and Spanish published databases. Behav. Res. Methods, Instrum. Comput. 32, 588–617 10.3758/BF03200832 11189860

[B9] Giard M. H. Peronnet F. (1999). Auditory-visual integration during multimodal object recognition in humans: a behavioral and electrophysiological study. J. Cogn. Neurosci. 11, 473–490 10.1162/089892999563544 10511637

[B10] Hocking J. Price C. (2008). The influence of colour and sound on neuronal activation during visual object naming. Brain Res. 1241, 92–102 10.1016/j.brainres.2008.08.037 18789907 PMC2693529

[B11] Hubbard T. L. (2010). Auditory imagery: empirical findings. Psychol. Bull. 136, 302–329 10.1037/a0018436 20192565

[B12] Humphreys G. W. Lamote C. Lloyd-Jones T. J. (1995). An interactive activation approach to object processing: effects of structural similarity, name frequency, and task in normality and pathology. Memory 3, 535–586 10.1080/09658219508253164 8574877

[B13] Kiefer M. Barsalou L. W. (2013). Grounding the human conceptual system in perception, action, and internal states, in Action Science: Foundations of an Emerging Discipline, eds Prinz W. Beisert M. Herwig A. (Cambridge, MA: MIT Press), 381–407

[B14] Kiefer M. Sim E.-J. Herrnberger B. Grothe J. Hoenig K. (2008). The sound of concepts: four markers for a link between auditory and conceptual brain systems. J. Neurosci. 28, 12224–12230 10.1523/JNEUROSCI.3579-08.2008 19020016 PMC6671691

[B15] Laurienti P. J. Wallace M. T. Maldjian J. A. Susi C. M. Stein B. E. Burdette J. H. (2003). Cross-modal sensory processing in the anterior cingulate and medial prefrontal cortices. Hum. Brain Mapp. 19, 213–223 10.1002/hbm.10112 12874776 PMC6871917

[B16] Matheson H. E. White N. McMullen P. A. (2014). Testing the embodied account of object naming: a concurrent motor task affects naming artifacts and animals. Acta Psychol. 145, 33–43 10.1016/j.actpsy.2013.10.012 24291119

[B17] Mulatti C. Ceccherini L. Coltheart M. (2014). What can we learn about visual attention to multiple words from the word-word interference task? Mem. Cognit. [Epub ahead of print]. 10.3758/s13421-014-0450-x 25052252

[B18] Mulatti C. Coltheart M. (2012). Picture-word interference and the response-exclusion hypothesis. Cortex 48, 363–372 10.1016/j.cortex.2011.04.025 21632048

[B19] Mulatti C. Lotto L. Peressotti F. Job R. (2010). Speed of processing explains the picture - word asymmetry in conditional naming. Psychol. Res. 74, 71–81 10.1007/s00426-008-0182-2 19002713

[B20] Nyberg L. Habib R. McIntosh A. R. Tulving E. (2000). Reactivation of encoding-related brain activity during memory retrieval. Proc. Natl. Acad. Sci. U.S.A. 97, 11120–11124 10.1073/pnas.97.20.11120 11005878 PMC27158

[B21] Pezzulo G. (2011). Grounding procedural and declarative knowledge in sensorimotor anticipation. Mind Lang. 26, 78–114 10.1111/j.1468-0017.2010.01411.x22068584

[B22] Potter M. C. Faulconer B. A. (1975). Time to understand pictures and words. Nature 253, 437–438 10.1038/253437a0 1110787

[B23] Redmann A. FitzPatrick I. Hellwig F. Indefrey P. (2014). The use of conceptual components in language production: an ERP study. Front. Psychol. 5:363 10.3389/fpsyg.2014.00363 24808878 PMC4010786

[B24] Schneider T. Engel A. Debener S. (2008). Multisensory identification of natural objects in a two-way crossmodal priming paradigm. Exp. Psychol. 55, 121–132 10.1027/1618-3169.55.2.121 18444522

[B24a] Van Selst M. Jolicoeur P. (1994). A solution to the effect of sample size on outlier elimination. Q. J. Exp. Psychol. 47A, 631–650

[B25] Vermeulen N. Corneille O. Niedenthal P. M. (2008). Sensory load incurs conceptual processing costs. Cognition 109, 287–294 10.1016/j.cognition.2008.09.004 18996513

[B26] Vredeveldt A. Hitch G. J. Baddeley A. D. (2011). Eye-closure helps memory by reducing cognitive load and enhancing visualisation. Mem. Cogn. 39, 1253–1263 10.3758/s13421-011-0098-8 21491166

