# Supplementary material for: The role of the sound of objects in object identification: evidence from picture naming
Source: Front Psychol. 2014 Oct 8;5:1139. doi: 10.3389/fpsyg.2014.01139 (PMC4189375; doi:10.3389/fpsyg.2014.01139)
Supplement: Supplementary file 1 [file DataSheet1.DOCX]

Appendix

Objects with a typical sound:

aereo (airplane), ambulanza (ambulance), ape (bee), armonica (harmonica), arpa (harp), aspirapolvere (vacuum), automobile (car), banjo (banjo), batteria (drums), bomba (bomb), bus (bus), camion (truck), campana (bell), cane (dog), cannone (cannon), carro armato (tank), cembalo (cymbal), cerniera (zipper), chitarra (guitar), cornamusa (bagpipe), doccia (shower), elicottero (helicopter), fisarmonica (accordion), fischietto (whistle), flauto (flute), flipper (pinball), frullatore (mixer), fucile (gun), fuoco (fire), gallo (rooster), giradischi (recordplayer), locomotiva (locomotive), lupo (wolf), macchina da cucire (sewingmachine), macchina da scrivere (typewriter), maracas (maracas), mitra (light machine gun), mosca (fly), motocicletta (motorcycle), mucca (cow), nave (boat), organo (organ), pianoforte (piano), piatti (music cymbals), pioggia (rain), pistola (gun), radio (radio), rasoio (razor), razzo (rocket), registratore (taperecorder), rubinetto (faucet), sax (saxophone), sveglia (clock), tamburo (drum), telefono (telephone), televisore (tv), tosaerba (lawnmower), tram (tram car), trapano (drill), trattore (tractor), treno (train), tromba (trumpet), trombone (trombone), ventilatore (fan).

Object without a typical sound:

aglio (garlic), ago (needle), albicocca (apricot), amo (hook), anguria (watermelon), aquilone (kite), asparago (asparagus), banana (banana), bicchiere (glass), binocolo (binocular), bottone (button), broccoli (sprouting broccoli), bussola (compass), cactus (cactus), calzino (sock), camicia (shirt), candela (candle), cappello (hat), caraffa (pitcher), carciofo (artichoke), carota (carrot), castagna (chestnut), ciliegia (cherry), cipolla (onion), falce (sickle), fiocco (bow), foglia (leaf), fragola (strawberry), fungo (mushroom), ghianda (acorn), giacca (jacket), girasole (sunflower), igloo (igloo), imbuto (funnel), letto (bed), libreria (bookcase), limone (lemon), lumaca (snail), maglione (sweater), mela (apple), melanzana (egg-plant), mora (mulberry), noce (walnut), palma (palm tree), panchina (bench), pantaloni (pants), papavero (poppy), pennello (paintbrush), peperone (pepper), pera (pear), pesca (peach), pettine (comb), pipa (pipe), piramide (pyramid), piselli (peas), piuma (feather), poltrona (armchair), pomodoro (tomato), sciarpa (scarf), secchio (bucket), sedano (celery), tavolozza (paint), uva (grapes), zucca (pumpkin).
